# Coexistence of *Pseudomonas aeruginosa* With *Candida albicans* Enhances Biofilm Thickness Through Alginate-Related Extracellular Matrix but Is Attenuated by N-acetyl-l-cysteine

**DOI:** 10.3389/fcimb.2020.594336

**Published:** 2020-11-24

**Authors:** Pornpimol Phuengmaung, Poorichaya Somparn, Wimonrat Panpetch, Uthaibhorn Singkham-In, Dhammika Leshan Wannigama, Tanittha Chatsuwan, Asada Leelahavanichkul

**Affiliations:** ^1^ Department of Microbiology, Faculty of Medicine, Chulalongkorn University, Bangkok, Thailand; ^2^ Center of Excellence in Systems Biology, Research Affairs, Faculty of Medicine, Chulalongkorn University, Bangkok, Thailand; ^3^ Translational Research in Inflammation and Immunology Research Unit (TRIRU), Department of Microbiology, Chulalongkorn University, Bangkok, Thailand

**Keywords:** biofilms, *Pseudomonas aeruginosa*, *Candida albicans*, l-cysteine, alginate

## Abstract

Bacteria and *Candida* *albicans* are prominent gut microbiota, and the translocation of these organisms into blood circulation might induce mixed-organism biofilms, which warrants the exploration of mixed- versus single-organism biofilms *in vitro* and *in vivo*. In single-organism biofilms, *Acinetobacter baumannii* and *Pseudomonas aeruginosa* (PA) produced the least and the most prominent biofilms, respectively. *C. albicans* with *P. aeruginosa* (PA+CA) induced the highest biofilms among mixed-organism groups as determined by crystal violet straining. The sessile form of PA+CA induced higher macrophage responses than sessile PA, which supports enhanced immune activation toward mixed-organism biofilms. In addition, *Candida* incubated in pre-formed *Pseudomonas* biofilms (PA>CA) produced even higher biofilms than PA+CA (simultaneous incubation of both organisms) as determined by fluorescent staining on biofilm matrix (AF647 color). Despite the initially lower bacteria during preparation, bacterial burdens by culture in mixed-organism biofilms (PA+CA and PA>CA) were not different from biofilms of PA alone, supporting *Candida*-enhanced *Pseudomonas* growth. Moreover, proteomic analysis in PA>CA biofilms demonstrated high AlgU and mucA with low mucB when compared with PA alone or PA+CA, implying an alginate-related mucoid phenotype in PA>CA biofilms. Furthermore, mice with PA>CA biofilms demonstrated higher bacteremia with more severe sepsis compared with mice with PA+CA biofilms. This is possibly due to the different structures. Interestingly, l-cysteine, a biofilm matrix inhibitor, attenuated mixed-organism biofilms both *in vitro* and in mice. In conclusion, *Candida* enhanced *Pseudomonas* alginate–related biofilm production, and *Candida* presentation in pre-formed *Pseudomonas* biofilms might alter biofilm structures that affect clinical manifestations but was attenuated by l-cysteine.

## Introduction

Biofilms are a community of microorganisms that grow on both nonliving and biotic surfaces to survive in harsh environments by producing multilayers of high-abundance extracellular matrix (ECM) consisting of proteins, polysaccharides, and nucleic acids (Flemming et al., 2007; de Kievit, 2009; Ghafoor et al., 2011; Flemming et al., 2016). Biofilms consist of 85% (by volume) matrix materials and 15% microbial cells (Flemming et al., 2007; Rasamiravaka et al., 2015). Surface-adherent (sessile) bacteria in biofilms become more antibiotic resistant than the free living (planktonic) form and result in recurrent infections (Darouiche et al., 2002; Aslam and Darouiche, 2011). Biofilms also possibly form nidus at the surface for the attachment of other pathogens that lead to biofilms of multiple bacteria or multiorganisms (Yang et al., 2011). The communication in mixed-organism biofilms is referred to as “quorum sensing” (Hossain et al., 2020) and might induce different biofilm properties than single-organism biofilms (Yang et al., 2011). Although catheter-related colonization of Gram-positive bacteria from skin microbiota (*Streptococcus* spp. and *Staphylococcus* spp.) is common, biofilms in the inner lumen of the catheter consist of both Gram-positive and -negative bacteria (Murga et al., 2001).

Both Gram-negative bacteria and *C. albicans* are the most and the second most predominant intestinal human microbiota, respectively, in which the natural interactions between these organisms is possible (Amornphimoltham et al., 2019). Because i) the translocation of gut microbiota (e.g., *Enterococcus* spp., Gram-negative bacteria, and *Candida albicans*) into blood circulation during sepsis (Alexandraki and Palacio, 2010; Amornphimoltham et al., 2019) and catheter-related candidiasis (Almirante et al., 2005; Tumbarello et al., 2012) are common, ii) mixed systemic infection between bacteria and *Candida* spp. is more severe than the infection by each organism separately (Bouza et al., 2013a; Bouza et al., 2013b; Dhamgaye et al., 2016), and iii) biofilms could be formed during bacteremia and fungemia (Shin et al., 2002; Li et al., 2018; Zheng et al., 2018); mixed-organism biofilms from bacteria and *Candida* spp. during sepsis are possible. Indeed, there is interaction between *Candida albicans* and Gram-negative bacteria, such as *Escherichia coli* (in peritonitis), *Pseudomonas aeruginosa* (in cystic fibrosis and ventilation-associated pneumonia), and *Acinetobacter baumannii* (in ventilation-associated pneumonia) (Dhamgaye et al., 2016). In addition, the observation of enhanced biofilm production has been mentioned with preliminary crystal violet staining (El-Azizi et al., 2004; Mear et al., 2013). Gut leakage–induced *Candida* translocation during sepsis (Leelahavanichkul et al., 2016; Amornphimoltham et al., 2019) might induce biofilms of bacterial-fungal collaboration, which result in persistent, recurrent, or severe infection (Chen and Wen, 2011; Wu et al., 2015).

Because i) antibiotic resistance caused by biofilm is a current serious medical problem (Shahrour et al., 2019), ii) biofilm eradication (bacterial and fungi) is difficult, and iii) antimicrobial treatment without biofilm-removal results in recurrent or persistent infection (Gunn et al., 2016); interventions or drugs for biofilm prevention/eradication are necessary (O’Toole et al., 2015). Here, we explore the interaction between gut-derived bacteria and *C. albicans* *in vitro* and in a catheter-subcutaneous implantation mouse model with the exploration in macrophage responses and the anti-biofilm evaluation.

## Materials and Methods

### Animals and Animal Models

Animal care and use protocol was approved by the Institutional Animal Care and Use Committee of the Faculty of Medicine, Chulalongkorn University, Bangkok, Thailand, based on the National Institutes of Health (NIH), USA. Male, 8-week-old C57BL/6 mice from Nomura Siam International (Pathumwan, Bangkok, Thailand) were purchased.

### Organism Preparation

Due to the limitation on biofilm production of organisms from laboratory strains, gut-derived bacteria and *Candida albicans* were isolated from blood samples of patients from the King Chulalongkorn Memorial Hospital (Bangkok, Thailand) and the same strain of organisms was used in all of the experiments. The sample accession process was approved by the ethical institutional review board, Faculty of Medicine, Chulalongkorn University according to the Declaration of Helsinki with written informed consent.

### Biofilm Induction and Anti-Biofilms *In Vitro*

**Figure 1:**
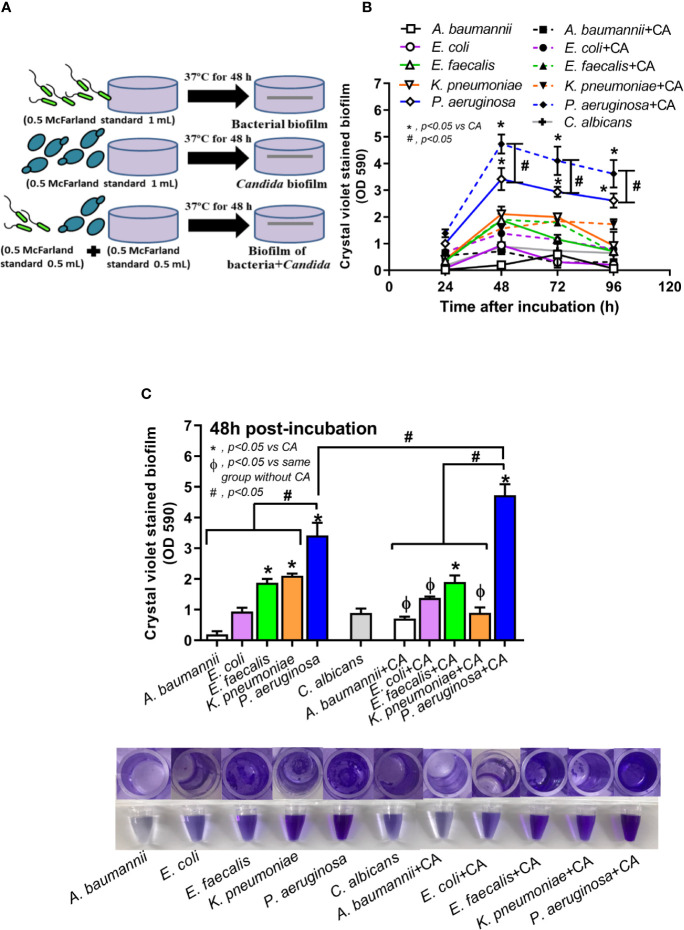
Diagram of the experimental design demonstrated lower bacteria in the biofilm preparation of mixed bacteria and *Candida* (bacteria + *C. albicans*) when compared with single-organism biofilms **(A)**, crystal violet–stained biofilm in 96-well plates from single- and mixed-organisms in the time course evaluation **(B)**, and in the biofilms at 48 h post-incubation with representative pictures of crystal violet stained 96-well plates and the color with acetic acid elution **(C)** are demonstrated. Mean ± SE was used for data presentation, and the differences between groups were examined for statistical significance by one-way ANOVA followed by Tukey’s analysis for comparisons of multiple groups or 2 groups **(B, C)**. A *p*-value of < 0.05 was considered statistically significant. (Independent triplicate experiments were performed.) (*p<0.05; ^#^ *p*<0.05).

To obtain the organisms in the early stationary phase that is suitable for biofilm production (Ricicova et al., 2010), bacteria and *C. albicans* were grown in Tryptic soy broth (TSB) (Oxoid Ltd., Basingstoke, Hampshire, England) and Sabouraud dextrose broth (SDB) (Oxoid), respectively, for 24 h at 37°C. Then, the samples were washed, resuspended in phosphate buffered saline (PBS; pH 7.4), and adjusted to the turbidity of 0.5 McFarland standard (approximately 1 × 10^8^ CFU/mL) in TSB. Then, the organisms were incubated at 37°C on 96-well plates (200 µL of TSB/well) for crystal violet staining and on microscope cover glasses (22x22 mm) (MenzelTM; Thermo Fisher Scientific, Waltham, MA, USA) or 25 mm segments of polyurethane catheter (NIPRO, Ayutthaya, Thailand) in 6-well plates (5 mL of TSB/well) for fluorescent staining. In the *P. aeruginosa* plus *C. albicans* group (*P. aeruginosa + C. albicans*), 0.5 mL of bacteria and *C. albicans* at 0.5 McFarland were combined into 1 mL for mixed-organism biofilm preparation, and 1 mL 0.5 McFarland was used for the preparation of single-organism biofilms (**Figure 1A**). Because *C. albicans* might be present on the previously formed bacterial biofilms, *C. albicans* administration after 24 h of *P. aeruginosa* incubation was performed (*P. aeruginosa* > *C. albicans*). The potential anti-biofilms l-cysteine (Sigma-Aldrich, St. Louis, MO, USA) (Olofsson et al., 2003; El-Baky et al., 2014) and β-defensin 3 (Peptide institute, Inc., Ibaraki-Shi, Osaka, Japan) (Batoni et al., 2016) in different concentrations were preliminary tested for bactericidal effect incubation with the organisms in TSB (Oxiod) for bacterial burdens at 24 h by optic density 600 (OD 600) with spectrophotometry (absorbance reader; BioTek, Winooski, VT, USA). In addition, the anti-biofilms property of both molecules was tested by 96-well plate biofilms with crystal violet staining. Moreover, l-cysteine at 50 mM was incubated with the organisms for the determination of biofilms on cover glasses, catheters in incubator, and catheters in mice.

### Biofilm Induction and Anti-Biofilms *In Vivo*

To determine *in vivo* biofilms, a mouse model of catheter subcutaneous-implantation following a previous publication was performed (Buret et al., 1991). In brief, 25-mm catheters (NIPRO) were incubated with organisms for 3 h at 37°C before washing with PBS and subcutaneously implanted onto the mouse flank on both sides under isoflurane anesthesis. In the *P. aeruginosa* > *C. albicans* group, *P. aeruginosa* were incubated with catheters for 1.5 h at 37°C before *C. albicans* administration and then further incubated for another 1.5 h before washing and inserting into mice. In the anti-biofilm test, l-cysteine (Sigma-Aldrich) at 50 mM was incubated together with the organisms before subcutaneous insertion in mice. At 48 h after catheter insertion, mice were sacrificed with sample collection (blood and catheters) by cardiac puncture under isoflurane anesthesia. For survival analysis, mice were observed for 96 h after the insertion. Renal injury (serum creatinine) and liver damage (serum alanine transaminase) were determined by QuantiChrom Creatinine Assay (DICT-500) (Bioassay, Hayward, CA, USA) and EnzyChrom Alanine Transaminase assay (EALT-100, BioAssay), respectively. Serum cytokines were determined by enzyme-linked immunosorbent assay (ELISA) (Invitrogen).

### Biofilm Visualization and Organism Burdens From Biofilms

In 96-well plates, crystal violet color was used for preliminary quantity estimation of biofilm ECM. Briefly, the supernatant in 96-well plates was discarded, stained with 0.1% crystal violet (200 µL) (Sigma-Aldrich) in water for 15 min, washed with water, and solubilized with 30% acetic acid (200 µL) (Sigma-Aldrich) in water before measurement with the absorbance reader (BioTek) with absorbance at 590 nm. For cover glasses and catheters after 48 h incubation, the samples were washed twice with PBS, fixed with 10% formaldehyde for 15 min, and stained for i) ECM by concanavalin AF647 (Invitrogen; Carlsbad, California, USA) at 50 μg/mL, ii) bacterial DNA with SYTO9 (Invitrogen) at 3.34 μM, and iii) fungi by calcofluor white (Sigma-Aldrich) at 1 μg/mL for 30 min in the dark before visualization by LSM 800 Airyscan confocal laser scanning microscope (CLSM; Carl Zeiss, Jena, Germany) and Plan-Apochromat (Celldiscoverer7 LSM900 Airyscan2) for cover glass and catheter biofilms, respectively. Fluorescent intensity was then analyzed by ZEN imaging software (Carl Zeiss). To determine the organism burdens from biofilms, the biofilms were dissolved in normal saline (1 mL) and thoroughly vortexed for 5 min and then processed as follows: i) directly incubated in TSB (Oxioid) for bactericidal burdens evaluated by the absorbance reader (BioTek) with optical density 600 (OD 600) at 24 h at 37°C incubation or ii) directly plated on tryptic soy agar (TSA) or Sabouraud dextrose agar (SDA) (Oxoid) in serial dilutions for bacterial and fungal burdens, respectively, from the samples with a mixture of bacteria and fungi before colony enumeration at 48 h at 37°C incubation. Of note, characteristics of colonies from bacteria and fungi were distinguishable on culture agar plates.

### Macrophage Cell-Line Experiments

RAW264.7, a mouse macrophage cell line, was used because immune activation toward organisms in a free-living form (planktonic cells) versus in biofilms (sessile form) might be different. As such, the sessile-form organisms were prepared in a cover-glass system and planktonic forms by culture in TSB. Then, both samples were heat inactivated at 65°C for 30 min before sonication with a high-intensity ultrasonic processor (VC/VCX 130, 500, 750) at 25% amplitude and centrifugation at 10,000 rpm for 5 min to separate the supernatant. Then, macrophages at 5x10^5^ cells/well were incubated with the supernatant for 24 h before the evaluation of cytokines by ELISA (Invitrogen) and macrophage polarization by polymerase chain reaction (PCR). Total RNA from macrophages was prepared by RNA easy mini-kit (Qiagen) and high-capacity reverse transcription assay (Applied Biosystems, Warrington, UK) on the Applied Biosystems 7500 Real-165 Time PCR System (Applied Biosystems) using the SYBR^®^ Green PCR Master Mix (Applied Biosystems). The relative quantitation normalized to β-actin (an endogenous housekeeping gene) by comparative threshold (delta-delta Ct) method (2-ΔΔCt) was demonstrated. The list of primers for PCR is presented in **Supplementary Table 1**.

### Determination of Bacterial Genes

Bacterial genes that associated with ECM were determined by PCR as previously published (Toyofuku et al., 2012). Briefly, the biofilms from cover glasses were scraped into 1 mL of TRIzol reagent (Invitrogen) and then purified RNA was treated with DNase I (Thermo Fisher Scientific). Single-stranded cDNA was synthesized from total RNA by random hexamer primers using reverse transcriptase (RevertAid First Strand cDNA, Thermo Fisher Scientific) and PCR performed as previously described. The relative quantitation normalized to the housekeeping *16S rRNA* gene with the comparative cycle threshold against the expression in the *P. aeruginosa* group was demonstrated.

### Proteomic Analysis

Proteins were precipitated with cold acetone from the equal volume of each condition, and the pellet was redissolved with urea (8 M) in 50 mM Tris‐HCl (pH = 8) to determine protein concentration by BCA protein assay (Thermo Fisher Scientific). Then, the soluble proteins were mixed with dithiothreitol (DTT), alkylated by iodoacetamide (IAA), digested with trypsin, added trifluoroacetic acid (TFA) to stop the digestion reaction, and dried by a SpeedVac centrifuge (Thermo Fisher Scientific). Subsequently, peptides were analyzed by the EASY-nLC1000 system coupled to a Q-Exactive Orbitrap Plus mass spectrometry (Thermo Fisher Scientific) at a flow rate of 300 nL/min with 5%–40% acetonitrile in 0.1% formic acid (FA) for 70 min followed by a linear gradient from 40%–95% acetonitrile in 0.1% FA for 20 min. Liquid chromatography mass spectrometer (LC-MS) analysis was performed using a 10 data-dependent acquisition method with an MS scan range of 350–1,400 *m/z* accumulated at a resolution of 70,000 full width at half maximum (FWHM) followed by a resolution of 17,500 FWHM. The normalized collision energy of higher energy collisional dissociation (HCD) was controlled at 32%. Precursor ions with unassigned charge state of +1 or those of greater than +8 were excluded, and the dynamic exclusion time was set to 30 s. Data acquisitions were monitored by Xcalibur 4.4 software (Thermo Fisher Scientific). Peptide spectrums were identified by SEQUEST-HT search engine against a mouse UniProt FASTA database. Searching parameters were i) digestion enzyme: trypsin, ii) maximum allowance for missed cleavages: 2, iii) maximum of modifications: 4, iv) fixed modifications: carbamidomethylation of cysteine (57.02146 Da), and v) variable modifications: oxidation of methionine (15.99491 Da). Biological process analysis of the host proteins was carried out using the PANTHER (http://www.pantherdb.org/) software program. Mass spectrometry proteomic data were determined and submitted to the ProteomeXchange consortium *via* the PRIDE (http://www.proteomexchange.org), and are available under the data set identifier PXD020949.

### Statistical Analysis

Mean ± standard error (SE) was used for data presentation, and the differences between groups were examined for statistical significance by one-way analysis of variance (ANOVA) followed by Tukey’s analysis or Student’s *t-*test for comparisons of multiple groups or 2 groups, respectively. Survival analysis was performed by Log-rank test. All statistical analyses were performed with SPSS 11.5 software (SPSS, IL, USA) and GraphPad Prism version 8.0 software (La Jolla, CA, USA). A *p*-value of < 0.05 was considered statistically significant.

## Results

### *Candida* Enhances *Pseudomonas* Biofilms and Increases Macrophage Responses

**Figure 2:**
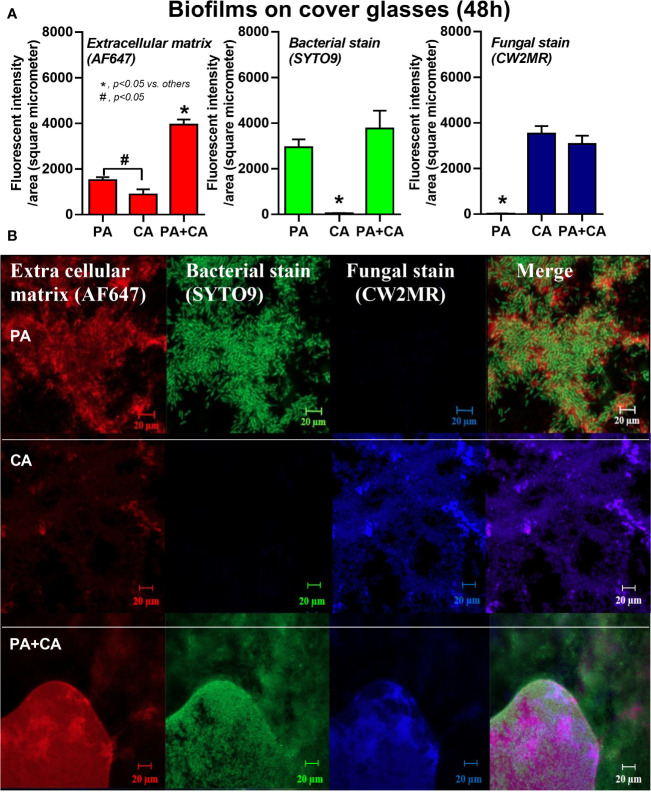
Intensity of fluorescent stains from 48 h biofilm on cover glasses of *P. aeruginosa* alone, *Candida* alone, and *P. aeruginosa* + *C. albicans* (*P. aeruginosa* + *C. albicans*) for ECM by AF647 (red color fluorescence), bacterial nucleic acid by SYTO9 (green color fluorescence), and fungal cell wall by calcofluor white (CW2MR; blue color fluorescence) **(A)** with the representative fluorescent images **(B)** are demonstrated. There was different depth on biofilms in images of the *P. aeruginosa* + *C. albicans* group because of the prominent biofilm thickness. Mean ± SE was used for data presentation, and the differences between groups were examined by one-way ANOVA followed by Tukey’s analysis for comparisons of multiple groups or 2 groups **(A)**. A *p*-value of < 0.05 was considered statistically significant. (Independent triplicate experiments were performed.) (*p<0.05; ^#^ *p*<0.05).

**Figure 3:**
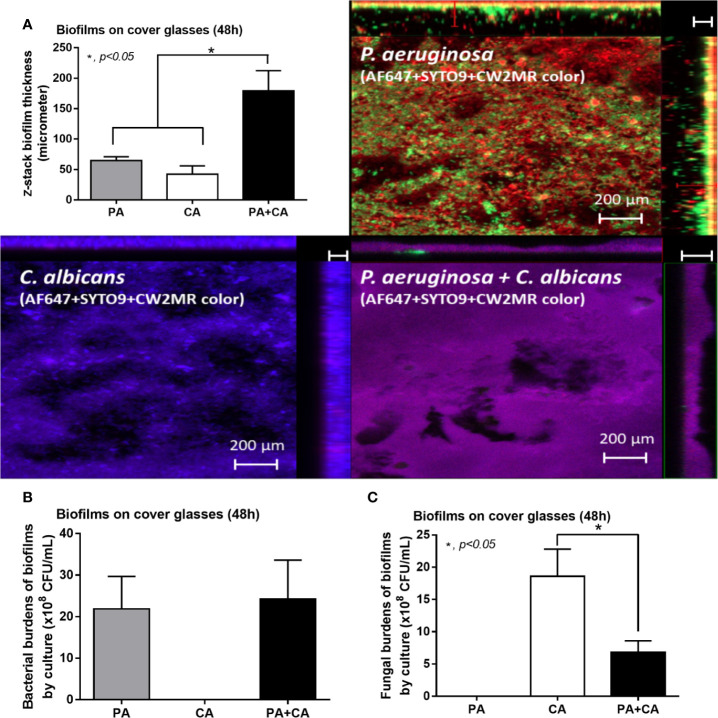
Biofilm thickness from 48 h biofilm on cover glasses of *P. aeruginosa* alone, *Candida* alone, and *P. aeruginosa* + *C. albicans* (*P. aeruginosa* + *C. albicans*) as evaluated by z-stack analysis of fluorescent images **(A)** and the burdens of organisms by culture for bacteria (TSA) and for fungi (SDA) **(B, C)** are demonstrated. Mean ± SE was used for data presentation, and the differences between groups were examined by one-way ANOVA followed by Tukey’s analysis for comparisons of multiple groups or 2 groups, **(A–C)**. A *p*-value of < 0.05 was considered statistically significant. (Independent triplicate experiments were performed.)

**Figure 4:**
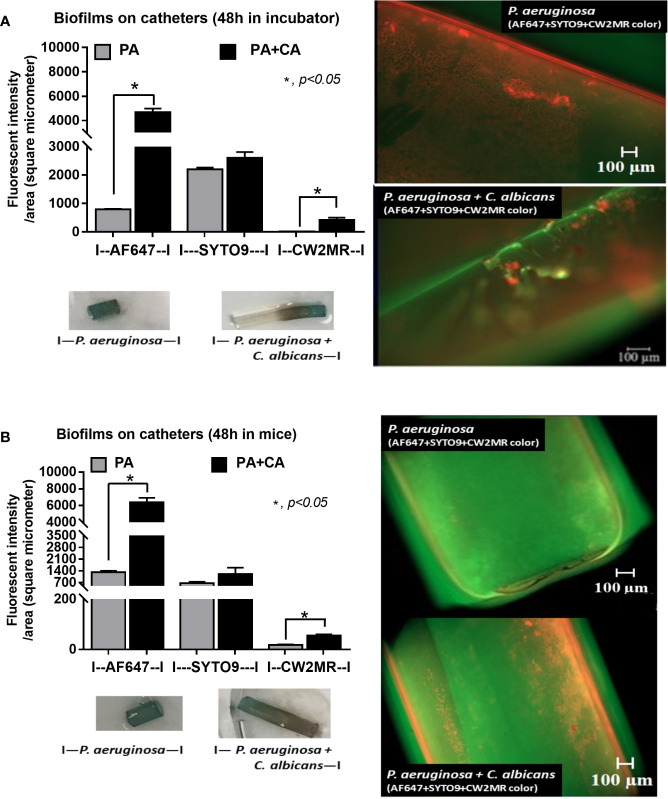
Diagram of the experimental design demonstrated the lower bacteria during biofilm preparation of *P. aeruginosa* and *C. albicans* (*P. aeruginosa* + *C. albicans*) compared with *P. aeruginosa* alone with the score of fluorescent intensity for ECM (AF647), bacterial nucleic acid (SYTO9), and fungal cell wall (CW2MR) together with representative fluorescent images (right side) with eye visualization biofilms (below) of the catheter biofilms after 48 h in an incubator **(A)** and in mice **(B)** are demonstrated. Mean ± SE was used for data presentation, and the differences between groups were examined by Student’s *t-*test for comparisons of multiple groups or 2 groups **(A, B)**. A *p*-value of < 0.05 was considered statistically significant. (Independent triplicate experiments were performed for A and *n* = 5/group for **B**.)

*Candida albicans* enhanced biofilm production of *P. aeruginosa* through the induction of alginate-related proteins and was attenuated by l-cysteine, which has been proposed as an interesting anti-biofilm in clinical situations. Crystal violet–stained biofilms were higher in *P. aeruginosa* plus *C. albicans* (PA+CA) as early as 48 h post-incubation (**Figure 1B**). Without *Candida*, *P. aeruginosa* (PA) produced more biofilms than other bacteria, and most bacteria, except *A. baumannii* and *E. coli*, produced more biofilms than *C. albicans* (CA) (**Figure 1B**). Despite the lower initial bacterial burdens, biofilms of mixed bacteria *Candida*, except *K. pneumoniae*, were similar to or higher than biofilms from bacteria alone (**Figure 1C**), implying an additive effect of fungi on biofilm production. Confocal electron microscope examination of PA+CA biofilms on cover glasses demonstrated the highest ECM (**Figures 2A, B**) and thickness (z-stack imaging) (**Figure 3A**) with a similar intensity of bacterial nucleic acid (**Figure 2A**) and bacterial culture (**Figure 3B**) when compared with biofilms from bacteria alone. However, fungal burdens in PA+CA were lower than CA alone (**Figure 3C**), which implies an impact of lower fungal burdens in PA+CA preparation (**Figure 1A**) or antifungal molecules of *Pseudomonas* (Kerr et al., 1999). For a closer representative of the clinical situation, biofilms were induced in catheters using incubator or subcutaneous implantation. PA+CA induced the highest ECM (**Figures 4A, B**), which is similar to the cover glass biofilms (**Figure 2**). Of note, there was a trend of higher PA burdens in PA+CA compared with PA alone (**Figures 4A, B**) despite the initially lower number of bacteria.

**Figure 5:**
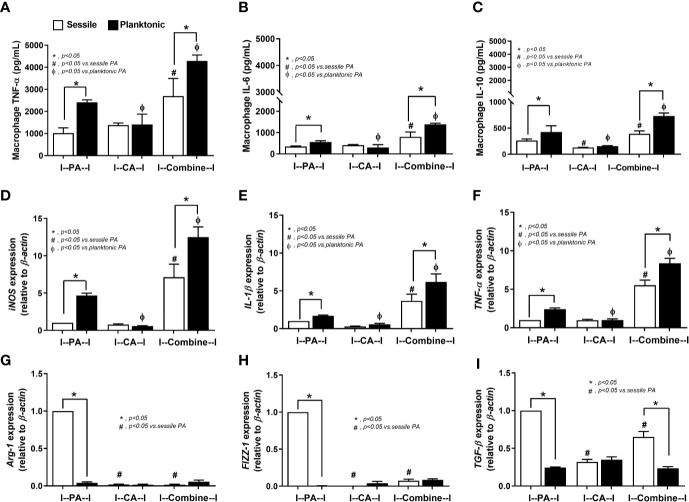
Characteristics of RAW264.7 cells (macrophages) after 6 h incubation of *P. aeruginosa*, *C. albicans*, or *P. aeruginosa* with *C. albicans* (combined) in sessile (biofilm) and planktonic form (free-living) as determined by supernatant cytokines (TNF-α, IL-6, IL-10) **(A–C)**, gene expression of macrophage polarization of M1 (*iNOS*, *IL-1β*, *TNF-α*) and M2 (*Arg-1*, *FIZZ-1*, *TGF-β*) **(D–I)** are demonstrated. Mean ± SE was used for data presentation, and the differences between groups were examined by one-way ANOVA followed by Tukey’s analysis for comparisons of multiple groups or 2 groups **(A–I)**. A *p*-value of < 0.05 was considered statistically significant. (Independent triplicate experiments were performed.) (*p<0.05; ^#^ *p*<0.05).

The responses against PA+CA biofilms were tested because the additive effect of *Candida* on bacterial immune responses was demonstrated in several models (Panpetch et al., 2017; Panpetch et al., 2019). With the planktonic (free-living) form of organisms (**Figure 5**, black columns), PA+CA induced the highest cytokine responses (TNF-α, IL-6, and IL-10) with the highest expression of genes for pro-inflammatory M1 macrophage polarization (*iNOS, IL-1β*, and *TNF-α*) and low expression of genes for anti-inflammatory M2 polarization (*Arg-1*, *FIZZ-1, TGF-β*) (**Figures 5A–I**). Planktonic PA induced higher cytokines and expression of M1 polarization genes with similar expression of M2 polarization genes when compared with planktonic CA (**Figures 5A–I**). In sessile (biofilm) form (**Figure 5**, white columns), PA+CA still induced the highest cytokines and gene expression of M1 polarization but in lower levels than the planktonic form (**Figures 5A–F**), which supports compromised antibacterial mechanisms of biofilms (Thurlow et al., 2011; Yamada and Kielian, 2019).

### *Candida* on the Pre-Formed *Pseudomonas* Biofilms Enhances Biofilm Thickness Through Matrix-Protein Induction, a Proteomic Analysis

**Figure 6:**
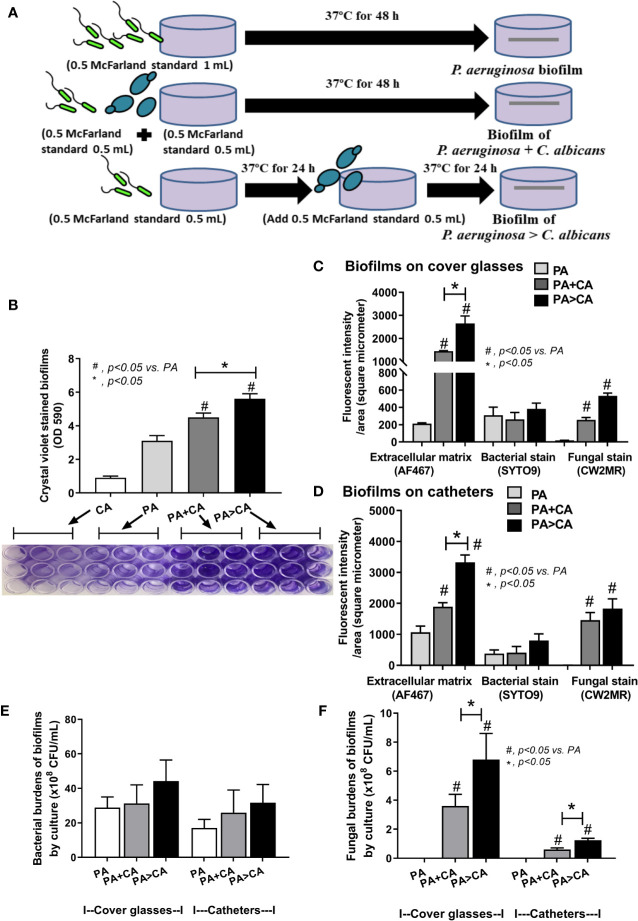
Diagram of the experimental design demonstrated lower bacteria in the biofilm preparation of simultaneous incubation of *P. aeruginosa* and *Candida* (*P. aeruginosa* + *C. albicans*) or the *Candida* addition in 24 h biofilm-formed bacteria (*P. aeruginosa* > *C. albicans*) in comparison with *P. aeruginosa* biofilms **(A)**, crystal violet–stained biofilm in 96-well plates with representative pictures **(B)**, fluorescent intensity of ECM by AF647 (red), bacterial nucleic acid by SYTO9 (green), and fungal cell wall by calcofluor white (CW2MR; blue) induced on cover glass biofilms and catheters (in incubator) **(C, D)** and organism burdens from biofilms using TSA for bacteria **(E)** and SDA for fungi **(F)** are demonstrated. Mean ± SE was used for data presentation, and the differences between groups were examined by one-way ANOVA followed by Tukey’s analysis for comparisons of multiple groups or 2 groups, respectively **(B–F)**. A *p*-value of < 0.05 was considered statistically significant. (Independent triplicate experiments were performed.) (*p<0.05; ^#^ *p*<0.05).

**Figure 7:**
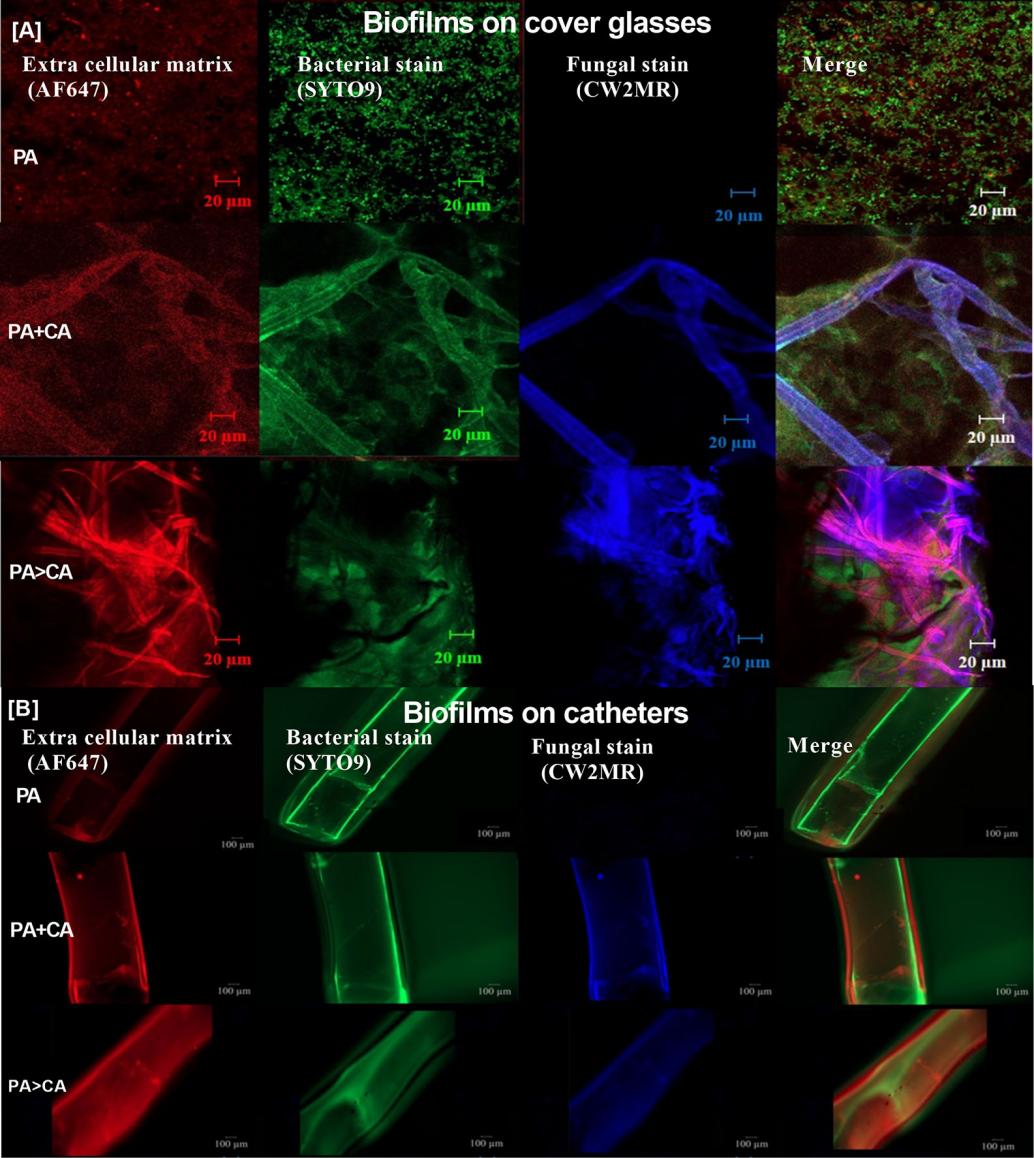
Representative fluorescent images of biofilms induced on cover glasses and catheters (in incubator) **(A, B)** stained for ECM by AF647 (red), bacterial nucleic acid by SYTO9 (green), and fungal cell wall by calcofluor white (CW2MR; blue) are demonstrated.

Because gut leakage–induced candidemia could be presented during bacterial sepsis (Amornphimoltham et al., 2019) and catheter-related bacterial sepsis is common (Phua et al., 2019; Reitzel et al., 2019), interaction between *Candida* and bacterial biofilm is possible. Then, *C. albicans* were added on *Pseudomonas* biofilms (*P. aeruginosa* > *C. albicans*; PA>CA) in comparison with biofilms that were formed with the initial mixture of organisms (PA+CA) (**Figure 6A**). Accordingly, PA>CA and PA+CA demonstrated higher biofilm formation compared with PA alone by crystal violet staining (96-well plates) (**Figure 6B**). Of note, fungal biofilms of CA alone were much less when compared with other groups in 96-well plate staining (**Figure 6B**), and the fungal biofilms were non-detectable in catheters (data not shown). Biofilms by fluorescent intensity of ECM (AF467) (cover glasses and catheters) were not different in bacterial burdens (SYTO9 stains and culture) (**Figures 6C–E** and **7**) despite the lower initial organisms in the preparation of PA>CA and PA+CA versus PA biofilms (**Figure 6A**). Of note, *Candida* burdens in PA>CA were higher than PA+CA biofilms by culture (**Figure 6F**) but not by fluorescent staining (**Figures 6C, D**). This is perhaps due to the higher nutrients in the culture system. Nevertheless, these imply the possible differences between biofilm structures of PA>CA versus PA+CA that lead to the proteomic analysis that focuses on *Pseudomonas* ECM proteins.

**Figure 8:**
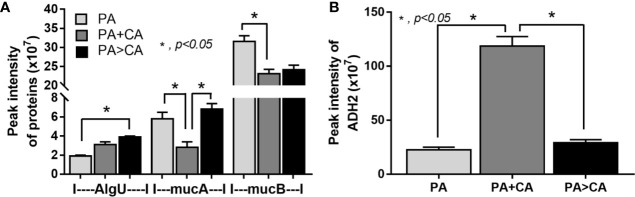
Proteomic analysis of biofilms from *P. aeruginosa* alone (PA) and PA with *Candida* by simultaneous incubation of *Candida* together with bacteria (*P. aeruginosa* + *C. albicans*; PA+CA) or the *Candida* addition in 24 h biofilm-formed bacteria (*P. aeruginosa* > *C. albicans*; PA>CA) as determined by the peak intensity of proteins in the ECM production process (AlgU, RNA polymerase sigma H factor; mucA, sigma factor AlgU negative regulatory protein; and mucB, negative regulator of the sigma factor AlgU) **(A)** and those of biofilms from *Candida albicans* alone (CA) and CA simultaneously incubated together with *P. aeruginosa* (*P. aeruginosa* + *C. albicans*; PA+CA) or the *Candida* addition in 24 h biofilm-formed bacteria (*P. aeruginosa* > *C. albicans*; PA>CA) as determined by the peak intensity of proteins in the ECM production process (ADH2, alcohol dehydrogenase) **(B)**. Mean ± SE was used for data presentation, and the differences between groups were examined by one-way ANOVA followed by Tukey’s analysis **(A, B)**. A *p*-value of < 0.05 was considered statistically significant. (Independent duplicate experiments were performed.)

Biofilms were demonstrated in all groups; 591 proteins from proteomic analysis that presented in all groups that are presented in the Venn diagram (**Supplementary Data 1**) were classified by protein biological process based on the PANTHER classification system (http://www.pantherdb.org) into the following: i) proteins in PA+CA and PA>CA that were higher than PA alone (37 proteins, including RNA polymerase sigma H factor; AlgU), ii) proteins in PA+CA that were higher than PA>CA (19 proteins without ECM proteins), iii) proteins in PA>CA that were higher than PA+CA (81 proteins, including sigma factor AlgU negative regulatory protein; mucA), and iv) proteins in PA+CA and PA>CA that were lower than PA (450 proteins, including negative regulator of the sigma factor AlgU; mucB) (**Supplementary data 1**). Most of bacterial proteins in PA+CA or PA>CA were lower than PA alone (**Supplementary data 1**) because of the lower initial bacteria in preparation of mixed-organism biofilms (**Figure 6A**). Indeed, high ECM initiators AlgU and mucA and low ECM inhibitor mucB (Schurr et al., 1996) in PA>CA compared with PA alone or PA+CA (**Figure 8A**) might be responsible for the prominent ECM in the PA>CA group. Of note, cleavage mucA is necessary for alginate production (Damron et al., 2009; Li et al., 2019). Likewise, the intercept proteins with *Candida* biofilms (CA) (**Supplementary data 2**) were divided into i) proteins in PA+CA and PA>CA that were higher than PA alone (4 proteins, including ADH2; alcohol dehydrogenase 2), ii) proteins in PA+CA that were higher than PA>CA (19 proteins without ECM proteins), and iii) proteins in PA+CA and PA>CA that were lower than PA (18 proteins without ECM proteins) (**Supplementary data 2**). Higher ADH2, an ECM restriction protein (Mukherjee et al., 2006), in PA+CA (**Figure 8B**) might be responsible for the lower ECM when compared with the PA>CA group (**Figures 6B–D**).

### Anti-Biofilms That Interfere With ECM Proteins *In Vitro* and *In Vivo* Evaluations

**Figure 9:**
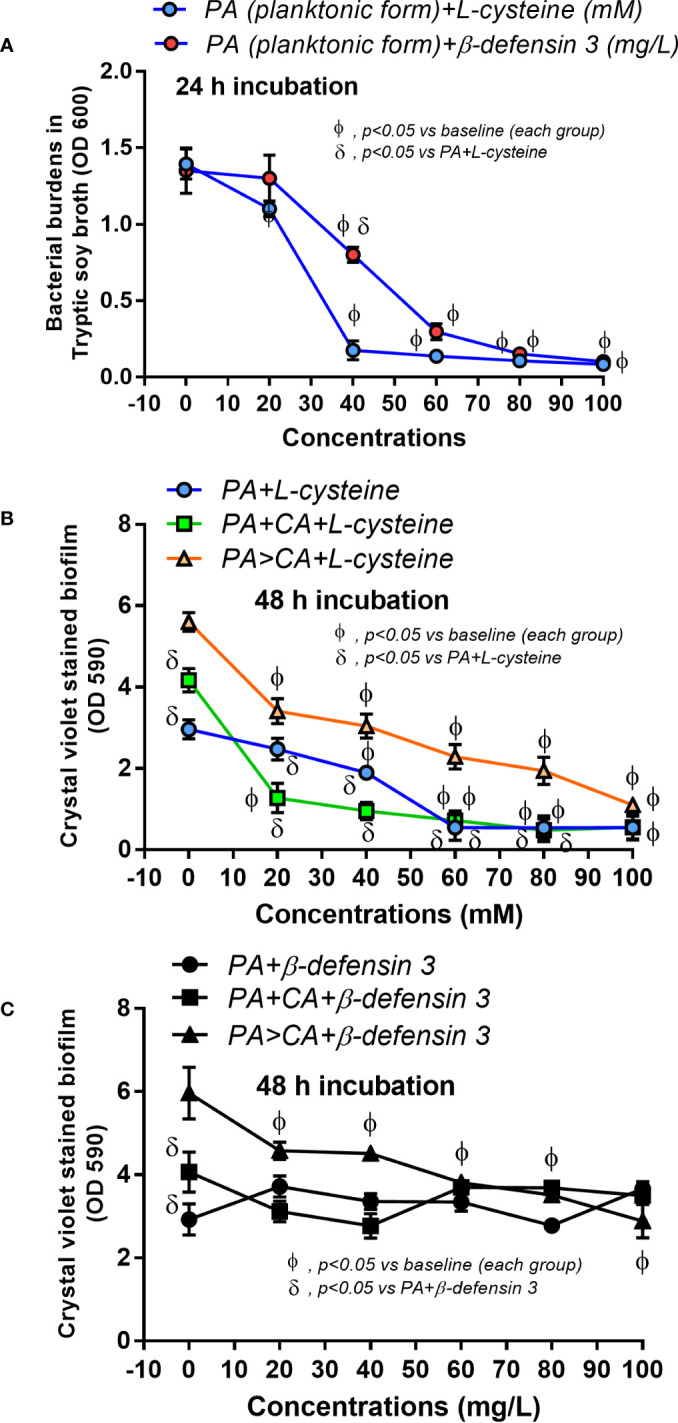
Effect of the different concentrations of l-cysteine and β-defensin 3 on bacterial burdens in TSB culture of planktonic *P. aeruginosa* after 24 h incubation **(A)** and crystal violet–stained biofilms on 96-well plates after 48 h incubation **(B, C)** are demonstrated. Mean ± SE was used for data presentation. The differences between groups were examined by Student’s *t-*test **(A)** and by one-way ANOVA followed by Tukey’s analysis **(B, C)**. The differences between the individual time points versus the baselines were examined by repeated-measures ANOVA analysis **(A–C)**. A *p*-value of < 0.05 was considered statistically significant. (Independent triplicate experiments were performed.)

**Figure 10:**
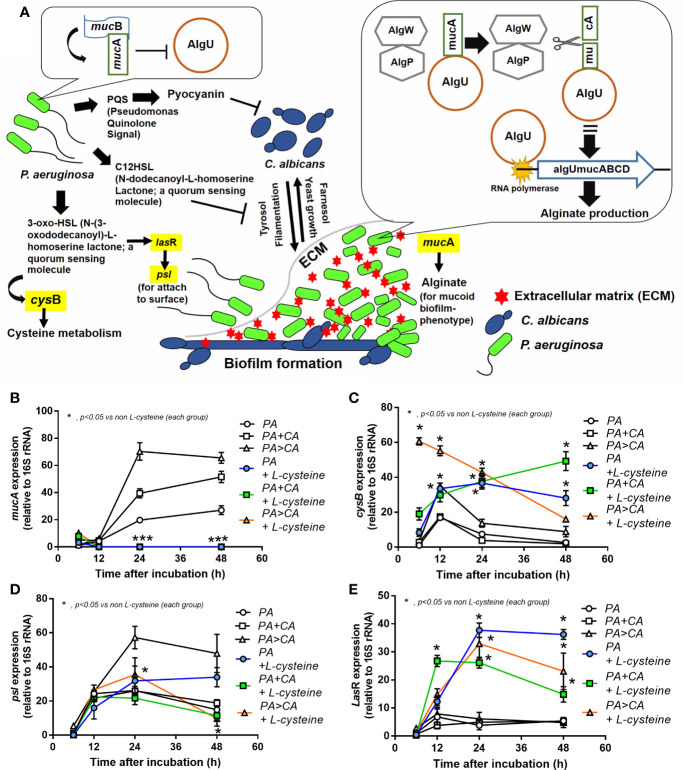
Diagram of some important genes that regulate ECM protein formation **(A)** demonstrates i) *Pseudomonas* produces 3-oxo-HSL and several proteins to initiate biofilms (yellow highlights are selected genes to explore in this study) and secretes pyocyanin and C_12_-HSL to inhibit *Candida* growth (Kerr et al., 1999), ii) *Candida* produces Tyrosol and Farnasol for fungal biofilms (Pierce, 2005; McAlester et al., 2008), iii) bacterial attachment on *Candida* pseudohyphae on mixed-organism biofilms (Fourie et al., 2016), and iv) biofilm inhibition by mucB in planktonic bacteria (left side) and alginate synthesis by AlgU in sessile bacteria (right side) (Schurr et al., 1996). Additionally, expression of some genes from biofilms of *P. aeruginosa* alone (PA) and PA with *Candida* by initially simultaneous incubation (*P. aeruginosa* + *C. albicans*) or the *Candida* addition at 24 h biofilm-formed bacteria (*P. aeruginosa* > *C. albicans*) with or without l-cysteine (anti-biofilm) **(B–E)** are demonstrated. Mean ± SE was used for data presentation, and the differences between groups were examined by one-way ANOVA followed by Tukey’s analysis for comparisons of multiple groups or 2 groups **(B–E)**. A *p*-value of < 0.05 was considered statistically significant. (Independent triplicate experiments were performed.) (*p<0.05; ***p<0.001).

**Figure 11:**
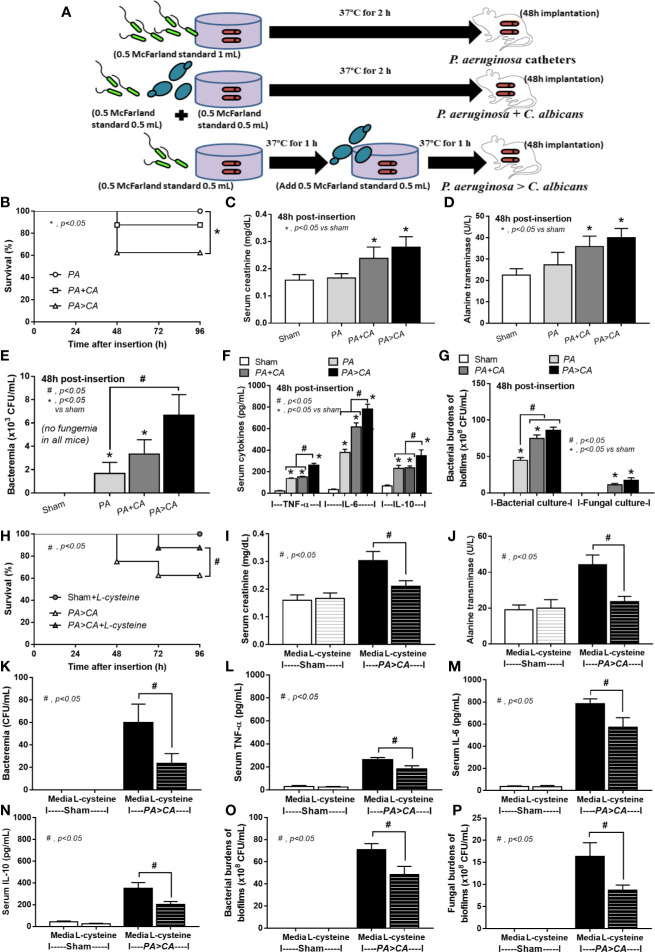
Diagram of the preparation of catheter biofilms of *P. aeruginosa* alone (PA) and with *Candida* by simultaneous incubation of *Candida* together with bacteria (*P. aeruginosa* + *C. albicans*) or the *Candida* addition in 1.5 h biofilm-formed bacteria (*P. aeruginosa* > *C. albicans*) before 48 h of subcutaneous implantation in mice **(A)** are indicated. Characteristics of the catheter insertion mouse model as determined by survival analysis **(B)**, kidney and liver injury (serum creatinine and alanine transaminase) **(C, D)**, bacteremia **(E)**, serum cytokines **(F)**, and organism burdens of catheter biofilms **(G)** are demonstrated. Additionally, characteristics of mice with *P. aeruginosa* > *C. albicans* biofilm catheters with or without l-cysteine incubation as evaluated by these parameters **(H–P)** are demonstrated. The survival analysis **(B, H)** and the differences between groups were examined by Log-rank test and one-way ANOVA followed by Tukey’s analysis, respectively **(C–G, I–P)**. A *p*-value of < 0.05 was considered statistically significant. (*n* = 12/group for survival analysis and *n* = 6–8/group for other parameters.)

Because i) biofilm ECM of *Pseudomonas* with *Candida* were more prominent than biofilms of *Pseudomonas* alone despite the lower bacteria in the preparation process (**Figures 2** **–** **4**, **6**, and **7**) and ii) proteins that accelerate ECM were demonstrated in the PA>CA group by the proteomic analysis (**Figures 8A, B**), anti-biofilms with ECM interference are of strong interest. Accordingly, l-cysteine, which is an ECM-reducing agent, and β-defensin 3, an anti-biofilm with bactericidal activity, reduced both planktonic PA and PA biofilms. However, only l-cysteine attenuated biofilms of PA+CA and PA>CA (**Figures 9A–C**). In addition, l-cysteine reduced gene expression of ECM promoters, including *muc*A, *cys*B, and *las*R but not *psl* (**Figures 10A–E**). To test the catheter biofilms *in vivo*, catheters with biofilms were prepared before subcutaneous implantation in mice (**Figure 11A**). Mice with biofilm catheters from PA>CA or PA+CA demonstrated more severe sepsis as determined by mortality, renal injury, liver damage, bacteremia, and serum cytokines (**Figures 11B–F**) with the higher burdens of *Pseudomonas* but not fungi in catheter biofilms (**Figure 11G**). Of note, bacterial burdens in cover glass and catheter biofilms (in incubator) were not different among PA, PA+CA, and PA>CA (**Figure 6E**) while bacterial burdens from catheter biofilms (in mice) of PA+CA and PA>CA were higher than PA alone (**Figure 11G**). This is possibly due to the higher organism nutrients in mice compared with the *in vitro* system.

**Figure 12:**
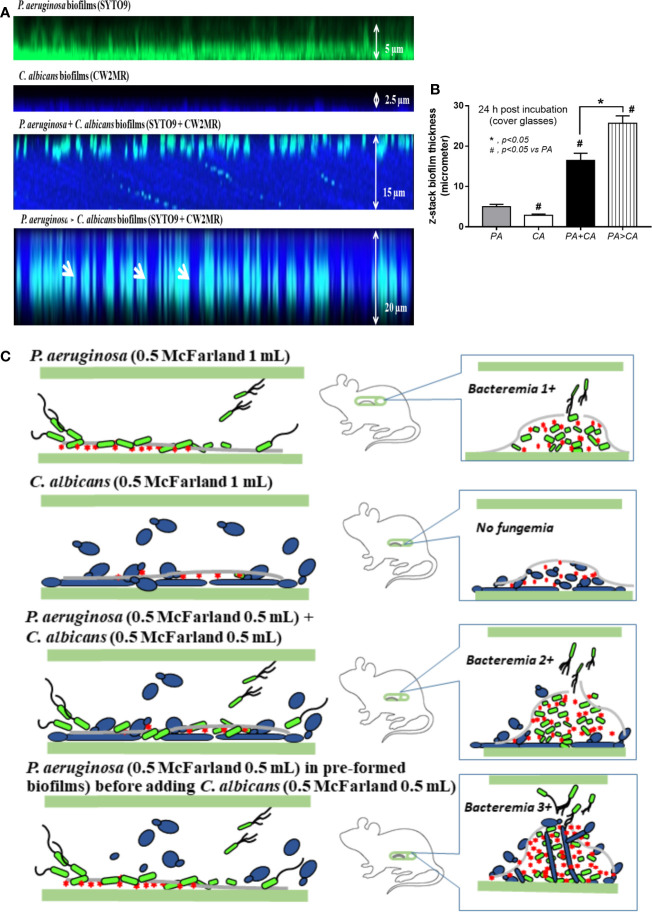
Representative fluorescent z-stack lateral-view images of bacterial nucleic acid by SYTO9 (green) and fungal cell wall by calcofluor white (CW2MR; blue) from biofilms of single (*P. aeruginosa* or *C. albicans*) and mixed organisms that initially incubated together and incubated for 24 h (*P. aeruginosa* + *C. albicans*) or the *Candida* addition at 12 h biofilm-formed bacteria (*P. aeruginosa* > *C. albicans*) and further incubated for another 12 h on cover glasses **(A)** is demonstrated (arrows indicate the possible *Candida* germ tubes that grow from the top into the bottom of biofilms). In addition, thickness of the biofilms **(B)** and the working hypothesis (left side, catheter during the preparation; right side, the proposed situations in catheter biofilms *in vivo*) show the enhanced bacterial dissemination in *P. aeruginosa* > *C. albicans* biofilms from *Candida* germ-tube elongation **(C)** are demonstrated. Mean ± SE was used for data presentation, and the differences between groups were examined by one-way ANOVA followed by Tukey’s analysis for comparisons of multiple groups or 2 groups **(B)**. A *p*-value of < 0.05 was considered statistically significant. (Independent triplicate experiments were performed for **A**, **B**.)

Bacteremia in PA>CA was most severe among all groups (**Figure 11E**), implying the easier detachment of bacteria from the biofilms compared with biofilms of PA alone. Because PA>CA demonstrated the most severe sepsis, biofilm catheters with or without l-cysteine were implanted into this model. As such, l-cysteine attenuated catheter-induced sepsis in the PA>CA group as determined by the previously mentioned sepsis parameters (**Figures 11H–N**) and reduced burdens of both bacteria and fungi in catheter biofilms (**Figures 11O, P**), suggesting an impact of bactericidal and fungicidal properties. In addition, the possible structural difference between PA+CA versus PA>CA biofilms was demonstrated with z-stack lateral view images of 24 h cover glass biofilms. As such, bacteria (green color of SYTO9) were on top of fungi (blue color of CW2MR) in PA+CA biofilms and vice versa on PA>CA with the prominent thickness in PA>CA biofilms (**Figures 12A, B**). The connections of blue color of fungi from the upper to the lower part of biofilms (**Figure 12A**, arrows) were possibly penetrating *Candida* germ-tube onto biofilms that induced bacterial exit-portals and caused easier bacterial dissemination in mice.

## Discussion

Although the enhanced *Pseudomonas* ECM production by the *Candida* presentation with the initial mixtures of both organisms are demonstrated (DeVault et al., 1990; Chen et al., 2014), the *Candida* presentation in the pre-formed *Pseudomonas* biofilms has not been studied. Indeed, adding *Candida* into pre-formed *Pseudomonas* bioflms induced a higher biofilm matrix partly through enhanced *muc*A expression with easier bacteremia when compared with mixed-organism biofilms from simultaneous incubation. In addition, l-cysteine, an anti-biofilm with a matrix inhibitor, attenuated mixed-organism biofilms both *in vitro* and *in vivo*.

Bacteremia or candidemia from gut-derived organisms is common (Amornphimoltham et al., 2019) and possibly increases risk of mixed-organism biofilms (Gominet et al., 2017). Although *Candida* produced less biofilm in most of the selected bacteria, perhaps due to the virulence (Hirota et al., 2017), *Candida*-enhanced biofilms of most bacteria, especially *P. aeruginosa* (PA), supported the cross-talk between these species (Fourie et al., 2016). In the intensive care unit, *P. aeruginosa* overgrowth in intestines (Okuda et al., 2010; Amornphimoltham et al., 2019), antibiotic-increased intestinal *C. albicans* (CA) (Panpetch et al., 2019), and mixed bacterial-fungal biofilms during sepsis (Pierce, 2005; McAlester et al., 2008) are mentioned. Here, PA+CA biofilms demonstrated prominent burdens of bacteria but not fungi compared with single-organism biofilms despite the lower organisms at preparation, which implies *Candida*-enhanced bacterial growth. Although immune responses against sessile organisms were lower than the planktonic forms (Yamada and Kielian, 2019), PA+CA in sessile forms induced more inflammatory effect than the sessile form of single organisms, supporting the sysnergistic responses against bacteria with fungi (Panpetch et al., 2017; Panpetch et al., 2019).

*Candida* on pre-formed *Pseudomonas* biofilms (PA>CA) induced a higher biofilm matrix than the simultaneous organism incubation (PA+CA) with similar bacterial burdens among PA, PA+CA, and PA>CA. The enhanced *Pseudomonas* growth in mixed-organism biofilms might, in part, be due to β-glucan, a major fungal cell wall component, as β-glucan mixed in culture media enhanced bacterial growth (Hiengrach et al., 2020) and oral β-glucan administration increased pathogenic bacteria in gut (Charlet et al., 2018). Likewise, mannans on *Candida* outer surface mediate β-glucosyltransferase binding, improving glucan-matrix production and enhancing bacterial–fungal association (Rodrigues et al., 2020). Although fungal burdens in PA>CA biofilms were higher than PA+CA biofilms, the impact of *Candida* on matrix production of mixed-organism biofilm was not dominant as *Candida* burdens of mixed-organism biofilms was lower than CA biofilms (**Figure 3C**). This was possibly due to the *Pseudomonas* antifungal effect (Kerr et al., 1999; Fourie et al., 2016).

*Pseudomonas* biofilms consist of at least 3 extracellular polymers, including i) polysaccharide synthesis locus (psl) for the cell attachment; ii) pel, a cationic exopolysaccharide for pellicle forming at the liquid interface; and iii) alginate for the mucoid phenotype that retains water (and nutrients) and provides resistance against antibiotics and host immunity (Lee and Yoon, 2017). Proteomic analysis in PA>CA demonstrated higher AlgU with lower mucB when compared with PA and higher mucA than PA+CA. As such, alteration of AlgU, mucA, and mucB implied the modification on alginate in the PA>CA group. Despite the possible lesser *Candida* impact on mixed-organism biofilms, ADH2, a biofilm-inhibitory enzyme (Mukherjee et al., 2006), in PA+CA was higher than PA>CA, which could possibly be associated with the lower biofilm matrix in PA+CA. Prominent ECM, but not organism burdens, should be a major component of mixed-organism biofilms; therefore, several genes for ECM synthesis were explored. As such, *muc*A and *psl* in PA>CA biofilms were higher than PA+CA and PA alone, and only *muc*A in PA+CA was higher than PA alone (**Figure 10**), implying the importance of *muc*A (anti-sigma factor) in mucoid biofilms of mixed organisms. Although mucA (an anti-sigma factor) binds to AlgU and inhibits its activity, cleavage-mucA provides an important step in matching with AlgU prior to AlgU release for alginate transcription (**Figure 10A**) (Damron et al., 2009; Li et al., 2019), and *muc*A mutation induces alginate overproduction (Moradali et al., 2017). Perhaps ethanol produced by *Candida* interferes with *muc*A gene expression as ethanol induces several molecules from *P. aeruginosa*, including mucA, exopolysaccharide (pel and psl), and antifungal phenazine [5-methyl phenazine-1-carboxylic acid (5MPCA)] (Chen et al., 2014; Tashiro et al., 2014). In addition, several other stimuli, such as NaCl and harsh environments, alter mucA and enhance biofilm formation (Harty et al., 2019). Hence, *Candida* presentation is an exacerbation factor of *Pseudomonas* biofilms, possibly through stress induction on bacteria. More studies on this topic are of interest.

Bacteremia and sepsis severity in mice with mixed-organism biofilms in catheters were more prominent than in mice with PA biofilms despite the initially lower bacteria at preparation (**Figure 11A**). In mixed-organism biofilms, bacterial burdens but not fungi were higher than PA biofilms *in vivo* (**Figure 11G**), and fungal burdens but not bacteria were higher than PA biofilms *in vitro* (**Figures 6E, F**). This demonstrated a proliferation ability of *P. aeruginosa* after *Candida* stimulation in an environment with less limitation on nutrients than the *in vivo* situation, which implies a possible complication of candidemia during *Pseudomonas* sepsis. On the other hand, synergy of *Candida* on *Pseudomonas* on biofilm production could be demonstrated both *in vivo* and *in vitro*. Because *Candida* induces *Pseudomonas* biofilm production, anti-biofilms with direct effect on biofilms are interesting. Here, N-acetyl-l-cysteine (l-cysteine), a thiol-containing cysteine derivative that disrupts disulfide bonds and cysteine utilization (Olofsson et al., 2003; Flemming et al., 2007), reduced ECM production in mixed-organism biofilms. In addition, bactericidal and fungicidal activity of l-cysteine (Olofsson et al., 2003; El-Baky et al., 2014) might also be responsible for reduced organism burdens inside catheters and attenuated septicemia. On the other hand, β-defensin 3, a bactericidal anti-biofilm, could not attenuate mixed-organism biofilms despite similar bactericidal activity with l-cysteine (Maisetta et al., 2006). Additionally, *Candida* enhanced alginate formation of *Pseudomonas*. *Candida* presentation in pre-formed *Pseudomonas* biofilms induced the thicker biofilms with an easier bacterial dissemination and could be possibly due to a defect on the biofilm surface during *Candida* germ-tube penetration (**Figure 12C**). Then, candidemia in patients with catheter-induced sepsis from *Pseudomonas* should be more clinically concerned, and l-cysteine is an interesting strategy against mixed-organism biofilms. However, our model of biofilm production was induced under static conditions. The utilization of a flow-cell system (Kerckhoven et al., 2016) should provide a more realistic condition. Further studies are interesting.

In conclusion, *C. albicans* on pre-formed *Pseudomonas* biofilms enhanced *Pseudomonas* ECM production through increased alginate producing genes, *AlgU* and *mucA*, and induced the easier biofilm disintegration that caused organismal dissemination. However, l-cysteine inhibited ECM production of the mixed-organism biofilms that might be useful for biofilm prevention and attenuation of catheter-related sepsis.

## Data Availability Statement

The mass spectrometry proteomics data have been deposited to the ProteomeXchange Consortium *via* the PRIDE [1] partner repository with the dataset identifier PXD020949.

## Ethics Statement

The animal study was reviewed and approved by The Institutional Animal Care and Use Committee of the Faculty of Medicine, Chulalongkorn University.

## Author Contributions

All authors listed have made a substantial, direct, and intellectual contribution to the work and approved it for publication.

## Funding

This study was supported by Thailand Government Fund (RSA-6080023), Thailand Research Fund (RES_61_202_30_022) and Ratchadaphiseksomphot Endowment Fund 2017 (76001-HR). PP was supported by Second Century Fund (C2F) for Postdoctoral Fellowship, Chulalongkorn University.

## Conflict of Interest

The authors declare that the research was conducted in the absence of any commercial or financial relationships that could be construed as a potential conflict of interest.
