# Supplementary material for: Coexistence of Pseudomonas aeruginosa With Candida albicans Enhances Biofilm Thickness Through Alginate-Related Extracellular Matrix but Is Attenuated by N-acetyl-l-cysteine
Source: Front Cell Infect Microbiol. 2020 Nov 24;10:594336. doi: 10.3389/fcimb.2020.594336 (PMC7732535; doi:10.3389/fcimb.2020.594336)
Supplement: Supplementary file 1 [file DataSheet_1.pdf]

Supplementary Table 1. List of Primers are demonstrated.

Primer names	genes	Forward	Reverse
Arginase-1	*Arg-1*	5'-CTTGGCTTGCTTCGGAACTC-3'	5'-GGAGAAGGCGTTTGCTTAGTTC-3'
Inducible nitric oxide synthase	*iNOS*	5'-ACCCACATCTGGCAGAATGAG-3'	5'-AGCCATGACCTTTCGCATTAG-3'
Interleukin-1β	*IL-1β*	5'-GAAATGCCACCTTTTGACAGTG-3'	5'-TGGATGCTCTCATCAGGACAG-3'
Tumor necrosis factor-α	*TNF-a*	5'-CCTCACACTCAGATCATCTTCTC-3'	5'-AGATCCATGCCGTTGGCCAG-3'
Resistin-like molecule-α	*FIZZ-1*	5'-GCCAGGTCCTGGAACCTTTC-3'	5'-GGAGCAGGGAGATGCAGATGAG-3'
Transforming growth factor	*TGF-β*	5'-CAGAGCTGCGCTTGCAGAG-3'	5'-GTCAGCAGCCGGTTACCAAG-3'
β-actin	*β-actin*	5'-CGGTTCCGATGCCCTGAGGCTCTT-3'	5'-CGTCACACTTCATGATGGAATTGA-3'
Anti-sigma factor MucA	*mucA*	5'-GATCGCGACACCGAACTAAT-3'	5'-GATCGCGACACCGAACTAAT-3'
Transcriptional regulator CysB	*cysB*	5'-ATGAAGCTTCAGCAATTGCGCTATA-3'	5'-TCAGTAGACCGGCAGTTCGATGCC-3'
Protein pslA	*psl*	5'-ATGAACGCTCTGTTCGATTGTCCAC-3'	5'-TCAAGCACTTGCACAGCAGACCT-3'
Transcriptional regulator LasR	*lasR*	5'-GTAGTTGCCGACGATGAAG-3'	5'-GTAGTTGCCGACGATGAAG-3'
16S ribosomal RNA	*16S rRNA*	5'-ACGCAACTGACGAGTGTGAC-3'	5'-GATCGCGACACCGAACTAAT-3'
