# Supplementary figures and images for: Coexistence of Pseudomonas aeruginosa With Candida albicans Enhances Biofilm Thickness Through Alginate-Related Extracellular Matrix but Is Attenuated by N-acetyl-l-cysteine
Source: Front Cell Infect Microbiol. 2020 Nov 24;10:594336. doi: 10.3389/fcimb.2020.594336 (PMC7732535; doi:10.3389/fcimb.2020.594336)

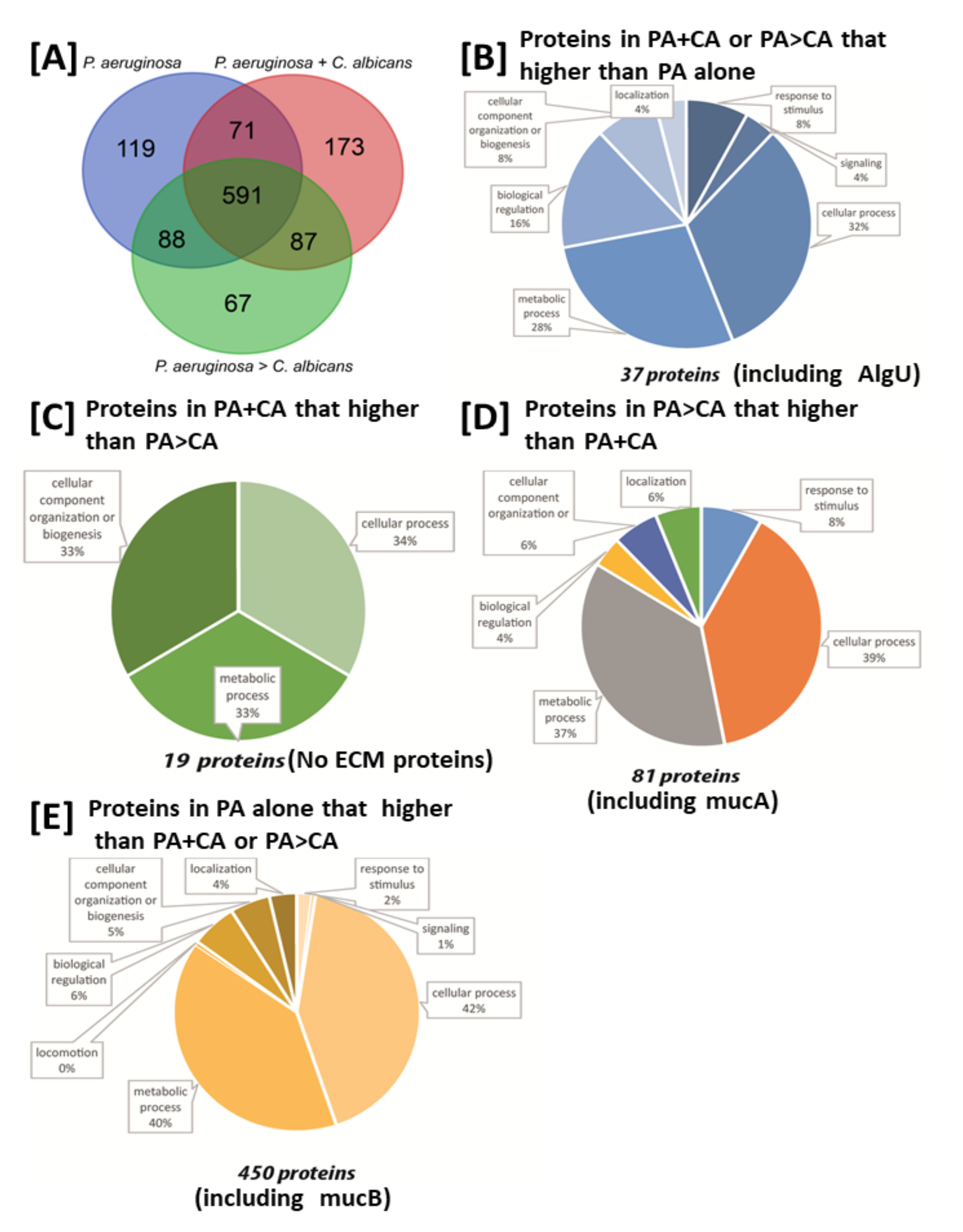

Supplement: Supplementary file 2 [file Image_1.tif]

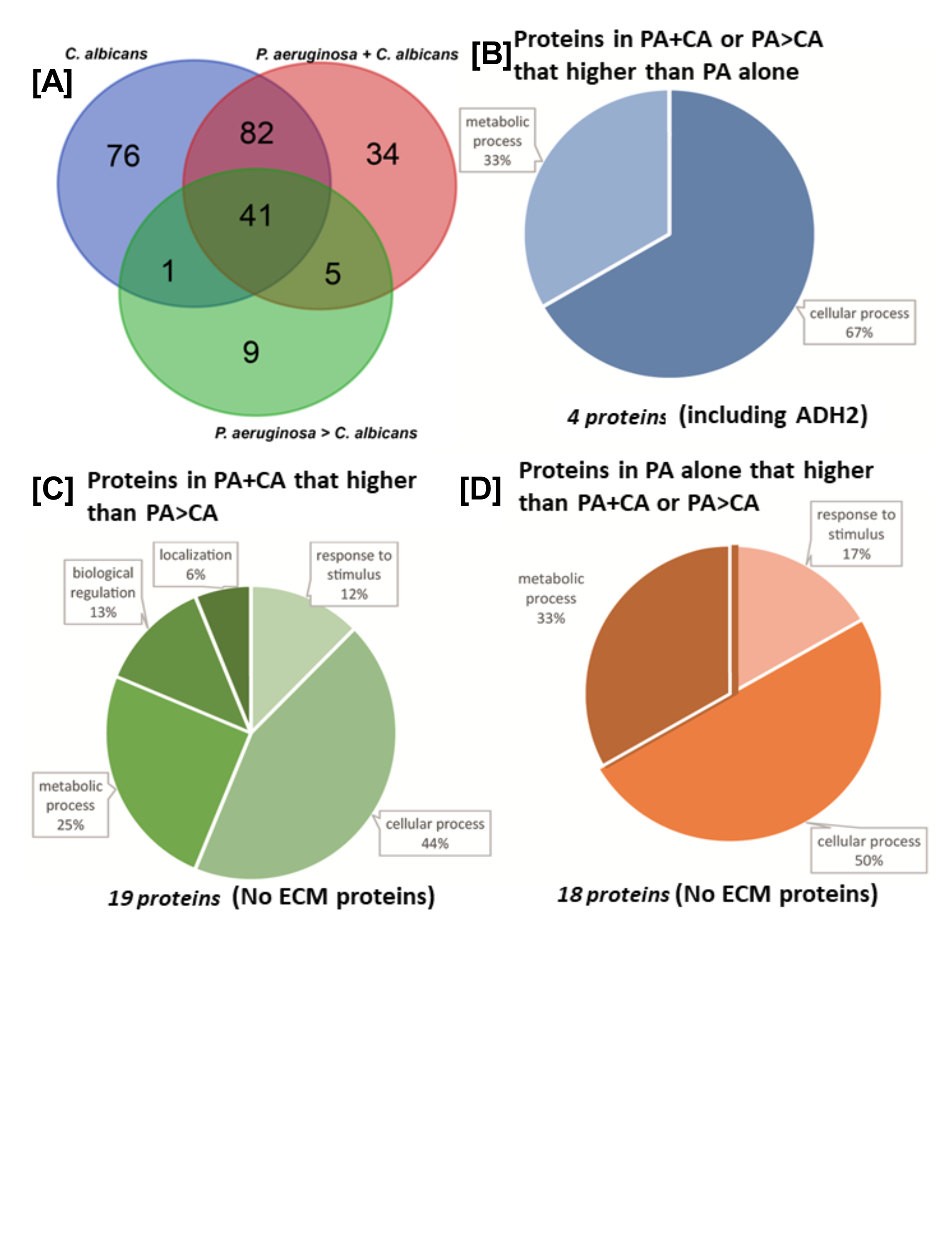

Supplement: Supplementary file 3 [file Image_2.tif]
